# Colorectal cancer screening among African American church members: A qualitative and quantitative study of patient-provider communication

**DOI:** 10.1186/1471-2458-4-62

**Published:** 2004-12-15

**Authors:** Mira L Katz, Aimee S James, Michael P Pignone, Marlyn A Hudson, Ethel Jackson, Veronica Oates, Marci K Campbell

**Affiliations:** 1 Lineberger Comprehensive Cancer Center, University of North Carolina at Chapel Hill, Chapel Hill, North Carolina, USA; 2 School of Public Health and The Ohio State University Comprehensive Cancer Center, Columbus, Ohio, USA; 3 Department of Internal Medicine, School of Medicine, University of North Carolina at Chapel Hill, Chapel Hill, North Carolina, USA; 4 Department of Nutrition, University of North Carolina at Chapel Hill, Chapel Hill, North Carolina, USA

## Abstract

**Background:**

A healthcare provider's recommendation to undergo screening has been shown to be one of the strongest predictors of completing a colorectal cancer (CRC) screening test. We sought to determine the relationship between the general quality of self-rated patient-provider communication and the completion of CRC screening.

**Methods:**

A formative study using qualitative data from focus groups and quantitative data from a cross-sectional survey of church members about the quality of their communication with their healthcare provider, their CRC risk knowledge, and whether they had completed CRC screening tests. Focus group participants were a convenience sample of African American church members. Participants for the survey were recruited by telephone from membership lists of 12 African American churches located in rural counties of North Carolina to participate in the WATCH (Wellness for African Americans Through Churches) Project.

**Results:**

***Focus Groups. ***Six focus groups (n = 45) were conducted prior to the baseline survey. Discussions focused on CRC knowledge, and perceived barriers/motivators to CRC screening. A theme that emerged during each groups' discussion about CRC screening was the quality of the participants' communication with their health care provider. **Survey**. Among the 397 participants over age 50, 31% reported CRC screening within the recommended guidelines. Participants who self-rated their communication as good were more likely to have been screened (36%) within the recommended guidelines than were participants with poor communication (17%) (OR = 2.8, 95% CI 1.2, 6.4; p = 0.013). Participants who had adequate CRC knowledge completed CRC screening at a higher rate than those with inadequate knowledge (p = 0.011). The percentage of participants with CRC screening in the recommended guidelines, stratified by communication and knowledge group were: 42% for good communication/adequate knowledge; 27% for good communication/inadequate knowledge; 29% for poor communication/adequate knowledge; and 5% for poor communication/inadequate knowledge.

**Conclusions:**

Participants who rated their patient-provider communication as good were more likely to have completed CRC screening tests than those reporting poor communication. Among participants reporting good communication, knowledge about colorectal cancer was also associated with test completion. Interventions to improve patient-provider communication may be important to increase low rates of CRC screening test completion among African Americans.

## Background

Colorectal cancer (CRC) screening among average risk adults 50 years and older can decrease the incidence and mortality rates for CRC [1-7]. National policy-making expert organizations recognize and support this evidence by recommending a variety of CRC screening testing strategies [8-13]. However, the 2001 Behavioral Risk Factor Surveillance System (BRFSS) reported only 23.5% of adults age ≥ 50 had fecal occult blood test (FOBT) within the last year, 38.7% had sigmoidoscopy or colonoscopy within the past 5 years, and only 53.1% of adults had been screened with either test within the recommended time periods. Data specifically from North Carolina are similar: 30% have had FOBT within 1 year, 31% have had sigmoidoscopy within 5 years, and 45% have had either test within the recommended time periods [14].

A healthcare provider's recommendation to undergo screening has been shown to be one of the strongest predictors of completing a CRC screening test [15-17], and has also been shown to be strongly correlated with initial and repeat mammography [18,19], and the completion of Pap smears[20]. Such recommendations may be more likely to occur when patients and providers communicate well, but previous research has not directly explored the relationship between patients' perceptions about the quality of patient-provider communication and the use of CRC screening. We sought to examine the association between perceived communication and CRC s screening in a sample of African-American church members in rural North Carolina.

## Methods

Data for this study were obtained as part of a larger study, the WATCH (Wellness for African Americans Through Churches) Project . The WATCH Project was a church-based colorectal cancer prevention study designed to increase fruit and vegetable consumption, reduce fat intake, increase moderate physical activity, and increase CRC screening among church members. In this study of patient-provider communication and CRC screening, we used data from two different sources: 1) focus groups of African American men and women, and 2) surveys of church members. The Institutional Review Board from the University of North Carolina at Chapel Hill approved this study.

### Focus groups

As part of the formative data collection in early 1998, we conducted six focus groups of African American adults. There were three focus groups of African American adult men and three of African American adult women. The focus groups comprised of a convenience sample drawn from members of African American churches located in central North Carolina. The focus groups were conducted in the churches, lasted approximately 60 minutes, were tape recorded, transcribed, and reviewed for accuracy. A $15 incentive and refreshments were provided for the participants. The focus groups were led by sex-matched African American moderators and explored issues associated with colon cancer, CRC screening (barriers and motivators), nutrition, and physical activity. The information from the focus groups was used to develop the questionnaires and to help guide the intervention strategies used in the culturally appropriate colon cancer prevention program.

### Survey

The second source of participants for the survey study was the baseline intervention sample for the larger WATCH project. The telephone survey collected information focused on general health status, nutrition, physical activity, patient-provider communication, CRC risk knowledge and screening behaviors. The baseline survey was completed, on average, in approximately 40 minutes and was administered by trained interviewers prior to the intervention. The baseline telephone surveys were conducted between October 1998 and October 1999.

In this study, a history of CRC screening was defined as the self-report of undergoing a fecal occult blood test (FOBT), flexible sigmoidoscopy, or colonoscopy within the recommended time period. Participants were asked whether they had each of the CRC screening tests, and if they responded yes, the participants were asked when they had their last test. The items in the survey included a brief explanation of each screening test. Items were described as follows: FOBT, "which is stool slides"; sigmoidoscopy, "which is a tube inserted in the rectum to look at the colon and the bowel"; and colonoscopy, "which is a tube inserted in the rectum to look at the entire intestine, usually given in a hospital or specialist's office." Responses included, "less than 1 year"; "1–2 years"; "3–5 years"; or "more than 5 years."

CRC screening was considered to be within the recommended time period based on an algorithm using ACS guidelines considering which test and how recent the test was performed (e.g. a person who reported having FOBT within the past year was considered within the recommended time period). Participants were considered to have been screened within the recommended time period if they had a FOBT within the preceding year, and sigmoidscopy within the preceding 5 years. Although current CRC screening guidelines recommend colonoscopy every 10 years for average-risk adults, we examined colonoscopy use in the past 5 years because of the limitations of the survey instrument. Self-report of CRC screening behavior has been demonstrated to be a reliable endpoint for intervention trials [22].

### Statistical analyses

Analyses of data from this study included factor analysis, analysis of variance, and logistic regression and were conducted using SPSS, version 10.1. Logistic regression analyses were performed to evaluate whether the level of perceived patient-provider communication was significantly related to CRC screening behavior in this population. Sociodemographic variables were identified as potential covariates if there was plausible theoretical or empirical evidence that the variable might be associated with the communication variable or with CRC screening. Variables that were significantly associated with communication level (p < 0.05) were retained and tested as covariates in the logistic models. Only the sex of the participant, receiving healthcare at a doctor's office versus a clinic/emergency room, and knowledge of CRC risk were significantly associated with communication and only these three covariates were entered into the initial logistic model. Variables in the model were evaluated by the Wald test and interpreted using odds ratios and confidence intervals. The overall fit of each model was evaluated using the Hosmer-Lemeshow Goodness of Fit test and by examining classification tables [23].

## Results

### Focus groups

Several major themes associated with CRC emerged from the personal experiences expressed about the medical community during the focus groups. One of the themes discussed by the participants in each focus group was patient-provider communication. Selected comments by focus group members about CRC and patient-provider communication are listed in Table 1. This important theme that emerged during the focus group was addressed in the baseline survey by adding items to the questionnaire that specifically addressed perceived communication with health care providers (Table 2).

**Table 1:** Focus groups (>50 years old): selected comments about patient-provider communication, colon cancer knowledge, and screening

"My doctor never even told me that I needed a digital or colonoscopy, or the technical term. The only concern is said was prostate. Nobody said anything about colon.'' [Male]
"I'm sixty years old and he's never told me to take one of these. I need to change doctors.'' [Female]
"Well you know, I think of men when I think of colorectal cancer.'' [Female]
"I've heard from 48 on up is a prostate exam more than anything else. I haven't heard anything about the colon. I really hadn't." [Male]

**Table 2:** Patient-provider communication scale*. Five Items**

A) I receive enough understandable information from my doctor/healthcare provider to make good decisions about my health.
B) I feel rushed during visits.
C) My doctor/healthcare provider involves me in decisions about my health care treatment.
D) I feel uncomfortable asking my doctor for tests or information if he/she doesn't mention it.
E) My doctor/healthcare provider understands my health needs.

*These items were included in the baseline survey because of the importance of the patient-provider communication theme that emerged from the focus group participants.

**Responses: Always, Almost always, Sometimes, Rarely, Never

Items A, C, and E loaded on the same factor (α = 0.74) and these three items were used in the final measure.

### Survey

Originally, 2480 names were obtained from the 12 church rosters located in five rural counties in North Carolina. Many members were ineligible (not 18 years old, deceased, no longer living in the state, medically incapable, phone number no longer working) or we were unable to contact them by telephone, and 239 members who declined to participate in the WATCH Project. There were 850 church members who participated in the WATCH Project and completed the baseline survey. The adjusted response rate was 66% using a calculation method, suggested by the Council of American Survey Organizations (CASRO) [21], that accounted for individuals whose eligibility and response status were unknown because program staff were never able to contact them.

The participants in this study were the 397 church members who participated in the WATCH Project and were 50 years and older. The characteristics of the 397 participants are shown in Table 3. Participants were mostly female (74%) and African American (98%). The mean age was 63 years (SD = 9.7). About half of the sample was currently married, 25% were widowed, and 14% were divorced. Thirty-seven percent had less than a high school education, 30% had a high school diploma or GED, 16% had some college or trade/beauty school, and 18% had a college degree or post-college education. Household income was answered by only 52% of the participants, and of the responders, 51% reported an income of less than $20,000.

**Table 3:** Communication and the characteristics of the participants ≥ 50 years old*

	Communication*				
	Total** n (%)	Good n (%)	Fair n (%)	Poor n (%)	F-test p-value
***Sex***					
Male	103(25.9)	71(23.8)	14(28.0)	18 (42.9)	F(2, 387) = 3.503
Female	293(73.8)	227(76.2)	36(72.0)	24 (57.1)	**p = .031**
***Education***					
Less than HS	145(36.5)	103(34.6)	21(41.2)	18 (42.9)	F(2, 388) = 0.409
HS/ GED	117(29.5)	91 (30.5)	14(27.5)	9 (21.4)	p = .665
Trade School/ College	135(34.0)	104(34.9)	16(31.4)	15 (35.7)	
***Income***					
<$20,000	184(51.0)	132(48.4)	26(57.8)	23 (59.0)	F(2, 354) = 1.134
$20,000–$49,999	128(35.5)	102(37.4)	12(26.7)	13 (33.3)	p = .323
≥ $50,000	49 (13.6)	39 (14.3)	7 (15.6)	3 (7.7)	
***Marital Status***					
Married	213(53.8)	164(55.2)	22(43.1)	24 (57.1)	F(2, 387) = 1.350 p=.261
Divorced/ Widowed/ Separated	162(40.9)	116(39.1)	26 (51.0)	18 (42.9)	
Never married	21 (5.3)	17 (5.7)	3 (5.9)	-----	
Healthcare Facility***					
Doctor's office	325(82.7)	250(85.0)	40(78.4)	29 (69.0)	F(2, 384) = 3.605
Clinic/ER/Health Dept.	68 (17.3)	44 (15.0)	11(21.6)	13 (31.0)	**p = .028**
Insurance****					
Medicaid/Medicare	176(44.3)	137(46.0)	19 (37.3)	19 (45.2)	F(2, 388) = 0.669 p = .513
No health insurance	21 (5.3)	14 (4.7)	4 (7.8)	3 (7.1)	F(2, 388) = 0.566 p = .568
Employer/self-paid	219(55.2)	167(56.0)	29 (56.9)	18 (42.9)	F(2, 388) = 1.344 p = .262

*Communication categories: the mean score for each category was calculated from the individual scores (continuous values)

** Numbers may not reflect the total n = 397 because of participants' refusals or missing data

***This variable is based on the questionnaire item: Where do you usually go when you need health care?

****Insurance variables were dichotomized for each category. Totals may exceed 100%, some respondents marked multiple categories (e.g., they had Medicare in addition to self-paid insurance).

Factor analysis of the five communication items was performed from the baseline survey responses and two factors were identified; one with three items and the other with two items. The second factor was dropped because it had only two items and did not add reliability to the scale. The three communication items about shared decision-making and patient satisfaction demonstrated good reliability (Cronbach's alpha = 0.74) and were summed to calculate a communication score. The communication score was used to categorize the participants into three groups: good, fair, and poor communication with providers. Participants were categorized as having "good" communication if they perceived receiving enough information from their provider, being involved in medical decisions, and thinking that their provider understood their health needs almost all the time or always. Participants who rated all three items 'sometimes', 'rarely', or 'never' scored "poor" on the communication scale, and individuals who rated the items with a mix of the above listed responses were assigned to the "fair" group.

In terms of quality of communication, 75% (298/397) responded positively to all 3 questions and were considered have "good" communication; 10% (42/397) responded positively to none of the 3 questions and were considered to have "poor" communication; and 13% (50/397) had fair results. Participants in the good communication group were more likely to be female (p = 0.031), and were more likely to receive their healthcare at a doctor's office versus a clinic/emergency room/health department (p = 0.028). None of the other sociodemographic factors listed in Table 3 appeared to vary significantly among communication groups. Participants categorized in the good communication group were more likely to report having been screened for CRC in the recommended time period compared to those in the poor communication group (35.9% vs. 16.7%; OR = 2.8, CI 1.2, 6.4, p = 0.013).

Only 45% (175/389) of the participants reported that their providers had recommended CRC screening, and just 31% (120/389) of all participants reported being screened within the recommended time interval. Of the individuals who reported being screened, 65% (78/120) stated that their doctor had recommended CRC screening, compared with 36% (97/269) of those who did not report screening.

Knowledge of CRC was assessed using seven items (Table 4) with a mean correct response of 3.8. If the participants answered at least four out of the seven items correctly, they were categorized as having adequate knowledge about colorectal cancer. The participants were considered to have inadequate CRC knowledge if they answered incorrectly or 'don't know' to ≥ 4 of the 7 items.

**Table 4:** Knowledge of colorectal cancer risk factors among 397 African American participants (≥50 years old) in the WATCH Project

Seven Items	Correct Answer	Percent*
1. A low fat and high fiber diet helps *decrease *colorectal cancer risk.	True	70.8%
2. The risk of colorectal cancer is *higher *in men than women.	False	13.6%
3. Physical activity *decreases *the risk for colorectal cancer.	True	42.6%
4. Colorectal cancer risk *increases *after age 50.	True	69.3%
5. A family history of colorectal cancer *does not *increase your risk.	False	49.1%
6. Finding cancer early *will not *increase the chances of surviving it.	False	65.7%
7. You *only *need to have a colorectal cancer screening test if you are having symptoms.	False	67.5%

*The percentage of participants who responded with the correct answer to each CRC knowledge item (n = 397)

Knowledge about CRC was considered adequate (knowledge score > = 4) for 57% (228/397) and inadequate for 43% (197/397). Participants with adequate CRC knowledge were more likely to have completed a CRC screening test within the recommended time period compared to those with inadequate CRC knowledge (21% vs. 10%). Adequate knowledge was associated with a higher level of education (p < 0.001), a higher level of income (p < 0.001), having health insurance (p < 0.001), and having Medicare/ Medicaid as one's health insurance (p < 0.001).

### Multivariate analyses

Results of the logistic regression analyses are shown in Table 5. Results were similar when using communication and CRC risk knowledge as continuous exposure variables, and when using a history of CRC screening anytime in the past as the outcome variable (instead of recent screening). For ease of interpretation, we chose to present the categorical analyses and use recent screening as the outcome of interest. After adjustment for the sex of the participant and source of healthcare, quality of communication remained significantly associated with completion of a CRC test.

**Table 5:** Factors associated with receiving CRC screening among 397 African American participants in the WATCH Project

Variable	OR (95% CI)	p-value
Sex	.65 (0.39, 1.07)	0.093
Source of healthcare (M.D. office vs. Clinic/ER)	1.07 (0.58, 1.95)	0.838
CRC Knowledge (Adequate vs. Inadequate)	1.82 (1.14, 2.89)	0.011
Patient-provider communication (Good vs. Poor/Fair)	1.95 (1.29, 2.94)	0.002

CRC screening within recommended guidelines by perceived communication and knowledge is listed in Table 6. The poor and fair communication groups were combined because of the small numbers within each category. Adequate knowledge is statistically significant for the good communication group but not for the fair/poor communication group. A test for interaction of communication and knowledge was performed for CRC within recommended guidelines and demonstrated no significant interaction.

**Table 6:** CRC screening results by communication and knowledge

	CRC screening in recommended time (%)	
Poor and Fair communication		
Inadequate knowledge	15.0	
(n = 40)		p = 0.654
Adequate knowledge	18.5	
(n = 54)		

Good communication		
Inadequate knowledge	27.4	
(n = 124)		p = 0.012
Adequate knowledge	41.6	
(n = 173)		

## Discussion

Our study found that participants had higher rates of CRC screening when their self-rated communication with their healthcare provider was classified as perceived as more positive. In addition, we found that participants who self-rated their communication as good and who had adequate CRC knowledge completed recent CRC screening at higher rates than those with good communication and inadequate knowledge. In addition, screening rates are higher with both good communication and adequate CRC knowledge than when only one factor is present. These findings suggest that both good patient-provider communication and CRC knowledge are important for CRC screening. Because of the cross-sectional nature of our study, we cannot determine causality, and it is possible that CRC knowledge improves by going through the screening process.

Improving CRC screening rates in the African American population may require strategies that address both improving physician-patient communication skills and increasing CRC knowledge. Good patient-provider communication is fundamental to a patient's perceived quality and satisfaction with their healthcare. Better communication, including the use of shared decision making, is associated with trust between patients and providers [24]. However, trusting the medical community remains a key concern for many African Americans. This is due, in part, to a long history of justifiable fear and mistrust of the medical and research communities stemming from the historical Tuskegee incident and other discriminatory practices in health care [25,26]. Our findings suggest that African Americans in this study generally had positive perceptions about communicating with health providers. Prospective data about communication and trust are needed to determine whether these perceptions predict greater screening compliance.

The association between the lack of physician recommendations for cancer screening tests and low patient utilization of those tests has been documented in previous investigations [18,19,27-30]. In a recent study of rural primary care practices, discussion about CRC screening occurred in only 14% of eligible patients [31]. Additionally, previous research has documented that African American patients receive less physician recommendations for cancer screening tests and utilize cancer screening tests at lower rates than other ethnic groups [27,32-34]. In another recent study of 150 African Americans (aged 50–79), 39% reported never having a recommendation for FOBT, 60% never had a flexible sigmoidoscopy recommended, and 57% never had a colonoscopy recommended [35]. In our study, only 45% of participants stated that CRC screening had been previously recommended by their healthcare provider.

The results of our study and other previously published work [24,35-39] suggest that CRC screening rates may improve by: 1) a focus on methods to improve patient-provider communication skills both for the patient and for providers; 2) addressing physician attitudes and behaviors toward recommending tests, and 3) providing patient CRC education that takes into account patients' literacy skills and preferred style of receiving information. Providing CRC risk knowledge and training to improve communication skills may be accomplished with various interventions [40]. Decision aids, systematically developed tools to provide information, increase knowledge, and encourage shared decision making, may also empower individuals to become more involved with their healthcare. Decision aids for CRC screening may be very useful because there is evidence that patients vary in their preference for how to be screened [36,37].

### Limitations

Although the results from our cross-sectional study have implications for developing colorectal cancer prevention programs, it must be recognized that prospective longitudinal studies are needed that specifically address the effect of patient-provider communication on CRC screening. Because of its cross-sectional design, we cannot infer causal relationships. In addition, since all participants were recruited from 12 churches, there may be some clustering of exposures and outcomes by physician that may affect the results; we did not measure this effect. Patient-provider communication was measured by using only three self-reported communication items, which may not fully capture the full extent of the patient-provider relationship. In addition, we do not know whether our participants had racially discordant or concordant physicians, and we did not have information regarding the physician's beliefs or practices about CRC screening or the physicians' assessment of the quality of communication with the patients. The outcome measure of having undergone CRC screening was self-reported, and these results were not validated. Finally, because of the relatively small number of participants and the use of convenience sampling, results from this study may not be generalizable.

## Conclusions

The findings from this cross-sectional study suggest that not only do patients need to be informed about their CRC risk and the importance of screening tests but that having good communication with their healthcare provider may also important. The burden of having good communication in the patient-provider relationship is the responsibility of both individuals. Both the patient and the provider should strive to improve their communication skills so that patients who want to participate in the decision about how to be screened for colorectal cancer may do so effectively.

Because culturally distinct factors may contribute to poor communication and mistrust for African-Americans, specific new strategies need to be developed [38]. Beliefs, attitudes, and concerns about cancer, prevention behaviors, and participation in medical decisions may not be the same for all races. If new prevention strategies for the African American population are developed, then there may be a chance at reducing the disparities associated with colon cancer and race.

## Competing interests

The author(s) declare that they have no competing interests.

## Authors' contributions

M.L.K. participated in the conception of the study, the analyses, the interpretation of the results, writing the first draft and revising the manuscript. A.S.J. participated in the conception of the study, performed the statistical analyses, participated in the interpretation of the results, and the editing of the manuscript. M.P.P. participated in the interpretation of the results and the writing of the manuscript. M.H., E.J., and V.O. assisted with the qualitative analysis and surveys. M.K.C. participated in the conception of the study, the statistical analyses, interpretation of the results, and the writing of the manuscript. All authors read and approved the final manuscript.

## Pre-publication history

The pre-publication history for this paper can be accessed here:


